# Effects of Hypoxia on Coral Photobiology and Oxidative Stress

**DOI:** 10.3390/biology11071068

**Published:** 2022-07-18

**Authors:** Mark Deleja, José Ricardo Paula, Tiago Repolho, Marco Franzitta, Miguel Baptista, Vanessa Lopes, Silvia Simão, Vanessa F. Fonseca, Bernardo Duarte, Rui Rosa

**Affiliations:** 1 MARE—Marine and Environmental Sciences Centre, Laboratório Marítimo da Guia and ARNET—Aquatic Research Infrastructure Network Associated Laboratory, Faculdade de Ciências da Universidade de Lisboa, Av. Nossa Senhora do Cabo, 939, 2750-374 Cascais, Portugal; jrpaula@fc.ul.pt (J.R.P.); tfrepolho@fc.ul.pt (T.R.); mfranzitta@fc.ul.pt (M.F.); msbaptista@fc.ul.pt (M.B.); vmlopes@fc.ul.pt (V.L.); silviazevedo95@gmail.com (S.S.); rarosa@fc.ul.pt (R.R.); 2 Division for Ecology and Biodiversity, Swire Institute of Marine Science, School of Biological Sciences, The University of Hong Kong, Pokfulam, Hong Kong SAR, China; 3 MARE—Marine and Environmental Sciences Centre and ARNET—Aquatic Research Infrastructure Network Associated Laboratory, Faculdade de Ciências da Universidade de Lisboa, Campo Grande, 1749-016 Lisboa, Portugal; vffonseca@fc.ul.pt (V.F.F.); baduarte@fc.ul.pt (B.D.); 4 Departamento de Biologia Animal, Faculdade de Ciências da Universidade de Lisboa, Campo Grande, 1749-016 Lisboa, Portugal; 5 Departamento de Biologia Vegetal, Faculdade de Ciências da Universidade de Lisboa, Campo Grande, 1749-016 Lisboa, Portugal

**Keywords:** climate change, hypoxia, *Acropora* spp., physiology, photochemistry, oxidative stress

## Abstract

**Simple Summary:**

With the growing importance of climate change research, the biological effects of oxygen loss on marine biota remain understudied. Coral reefs support diverse marine organisms and provide valuable ecosystem services. Deoxygenation may induce mass coral mortality and reduce species richness on coral reefs. Corals exposed to nocturnal hypoxia may experience detrimental effects due to induced oxidative stress caused by the increase in reactive oxygen species (ROS). The stress interferes with the most fundamental biological processes vital for the symbiosis between corals and photosynthetic algae. In this study, the coral *Acropora* spp. exhibited evident injury in photosynthetic apparatus, de-epoxidation state and DNA. However, besides that, there were no signs of engaged antioxidant defense mechanisms against ROS or pigment degradation, which leads to a conclusion that these corals may be resilient to such oxygen daily oscillations. Nevertheless, while corals might survive such short-term abiotic stress, the growing number and intensity of hypoxic events across the global ocean may pose a massive threat to these keystone invertebrate species.

**Abstract:**

Global ocean oxygen (O_2_) content is decreasing as climate change drives declines in oxygen solubility, strengthened stratification of seawater masses, increased biological oxygen consumption and coastal eutrophication. Studies on the biological effects of nocturnal decreased oxygen concentrations (hypoxia) on coral reefs are very scarce. Coral reefs are fundamental for supporting one quarter of all marine species and essential for around 275 million people worldwide. This study investigates acute physiological and photobiological responses of a scleractinian coral (*Acropora* spp.) to overnight hypoxic conditions (<2 mg/L of O_2_). Bleaching was not detected, and visual and physical aspects of corals remained unchanged under hypoxic conditions. Most photobiological-related parameters also did not show significant changes between treatments. In addition to this, no significant differences between treatments were observed in the pigment composition. However, hypoxic conditions induced a significant decrease in coral de-epoxidation state of the xanthophyll cycle pigments and increase in DNA damage. Although the present findings suggest that *Acropora* spp. is resilient to some extent to short-term daily oxygen oscillations, long-term exposure to hypoxia, as predicted to occur with climate change, may still have deleterious effects on corals.

## 1. Introduction

Dissolved oxygen is decreasing in the global ocean, with potential harmful impacts on diversity and abundance of marine organisms [1,2,3]. According to the Intergovernmental Panel on Climate Change (representative concentration pathway, RCP 2.6–8.5), the dissolved oxygen content in the global oceans will decline by 1.7% and 4% by 2100 as a result of general drivers of climate change [1,4]. Additionally, the oceans’ buffering capacity is diminishing due to increasing CO_2_ absorption and excess heat produced from anthropogenic activities [5,6,7,8]. The consequent ocean warming causes a reduction in oxygen solubility in seawater, which results in higher physiological oxygen demands of many marine organisms [3,9], with potential cascading effects (e.g., alteration of behavioural responses, migrations, reductions in growth rates and fecundity, and increased mortality rates) [3,10,11,12,13]. Oxygen concentrations in the range of 2 to 3.5 mg O_2_/L or below are considered as the hypoxic threshold for marine organisms [14,15,16,17]. Hypoxia is known to marine life at all levels of biological organization and induce stronger deleterious effects than ocean warming, ocean acidification, and their interaction [3,10]. Hypoxic or anoxic conditions appear in coastal zones and open oceans (known as oxygen minimum zones; OMZs) as a consequence of water column stratification, warming, organic matter production, increased rates of oxygen utilization, excessive agricultural run-off and limited circulation [18,19,20,21,22]. Coastal ecosystems are specially affected by increased warming waters and nutrient intrusion with more frequent marine heat waves and eutrophication. Therefore, the expansion of oxygen deprived coastal waters, specifically lethal acute hypoxic events, creates “dead zones” that lead to the occurrence of mass-mortalities of residing organisms, including corals [10,11,23,24,25,26,27].

Hermatypic scleractinian corals, the main reef-building species due to their calcium carbonate skeleton, have a mutualistic relationship with endosymbiotic dinoflagellate algae (Symbiodiniaceae), also termed zooxanthellae. Symbiodiniaceae are found in the hosts’ tissue and produce energy to meet most of scleractinian corals’ metabolic requirements via photosynthesis. Corals inhabit shallow, warm water areas with sufficient light, where they thrive in symbiosis [26,28,29,30,31]. Coral reefs cover one per cent of the global sea surface, yet they sustain many marine taxa by providing organisms with food, refuge, and substrate [30,31]. Perceptible lightening of the coral tissue defines coral bleaching, which can happen under multiple environmental conditions, including warming, acidification, algal blooms, pollution, and hypoxia [29,31,32,33,34]. This happens as the coral polyp expels the zooxanthellae, turning the colony into white or pale colours [35]. Marine hypoxia is known to impact corals’ health and can cause bleaching [10,36]. Global efforts to observe the impacts of hypoxia on coral reefs have only recently increased [3,24]. Corals conditioned to hypoxia for long-term periods are susceptible to severe bleaching resulting in benthic habitat degradation and mass mortalities of benthic and pelagic organisms [29,31,32,33,34,37].

Photo-oxidative stress, among a myriad of cellular, physiological and molecular responses, plays a major role in mass bleaching mortalities, which entails the production and accumulation of reactive oxygen species (ROS), following the oxidative theory of coral bleaching [37,38,39]. ROS are unstable molecules produced in the mitochondria of the coral-algal composite and principally in Symbodiniaceae chloroplasts [38]. Symbiodiniaceae are capable of overcoming antioxidant defence mechanisms in the coral cells and consequently restrict re-oxidation of reduced chemical species required for crucial elemental cycling [10]. The effects of ROS involve the inhibition of the photosynthetic and calcification rates, and impairment of metabolic pathways. Subsequently, this can prompt cellular damage and expulsion of symbionts to prevent physiological damage and potentially evade exocytosis, apoptosis of the host cells, separation of host cells and deterioration of the symbiotic cells through autophagy [38,39,40]. However, ROS are not solely responsible for initiating bleaching in corals since numerous cellular responses and diverse cell types are involved in the breakdown of the cellular functions and symbiont expulsion [39]. Hence, whether ROS initiates bleaching relies on the environmental perturbations and Symbiodiniaceae taxa [33]. Nonetheless, intracellular ROS production induces signaling cascades and can damage proteins, lipids and DNA if the organism fails to quench ROS accumulation that induces cellular oxidation [37].

The reaction pathways of antioxidant enzymes have the potential to remove ROS. The detoxification process to mitigate the effects of ROS begins with antioxidant defence reductive potential where superoxide dismutase (SOD) transforms superoxide anions (O_2_^−^) to hydrogen peroxide (H_2_O_2_). The latter is further reduced to non-toxic H_2_O and O_2_ by antioxidant enzyme catalase (CAT) [39,40,41,42]. The production of ROS in host and symbiont increases with lowered oxygen concentration, yet the complete antioxidant defence processes in host and symbionts remain unknown.

Evidence indicates that low DO concentration appears almost ubiquitously in coral reef landscapes. However, the diurnal fluctuations directed by respiration/consumption at night and photosynthetic oxygen production during the day create very complex conditions for the in situ benthos, with hypoxic conditions during the night and hyperoxic during the day. Hence, the severity of nocturnal hypoxia is extensively influenced by the biological activity of the associated benthos [15,37]. Moreover, nocturnal hypoxia is highly contingent on the tides and coral reef landscape, where entrapment of water and absence of water circulation (e.g., semi-enclosed lagoons, atolls and tidal pools) lead to night-time minimum O_2_ concentrations. Nocturnal hypoxia has been reported on coral reefs in the Red Sea [37,43]. The coral reefs of the Lizard and Heron Island lagoons in Australia demonstrated nocturnal O_2_ levels at around 20% or less due to oxygen consumption by organisms and photosynthesis absence [43,44]. Thus, shallow coral reefs are more inclined to exhibit daily oxygen depletion, in contrast to vast periodical or persistent ocean deoxygenation linked to ocean warming and anthropogenic nutrient intrusion. For instance, coral reefs in the Caribbean exhibited episodic anthropogenic hypoxia with lowest O_2_ content of around 0.5 mg O_2_/L, causing coral bleaching and mortality. Additionally, the northern Gulf of Mexico and East China Sea showed patterns of increasing hypoxic areas due to high nutrient loading [10,24,36,45,46,47]. Occurrence of nocturnal hypoxia is a more recurrent phenomena and higher threat as generally anticipated.

This study investigates, for the first time, the photochemical responses and antioxidant enzymatic defences of a scleractinian coral (*Acropora* spp.) to recurrent nocturnal hypoxic conditions (<2 mg/L of O_2_). More specifically, we evaluated the impacts of reduced oxygen levels during the night-time on: (i) survival, (ii) key photobiological parameters (e.g., absorption energy flux, electron transport energy flux, the density of the reaction centre II within the photosystem (PS) II antenna chlorophyll bed), (iii) pigment concentrations (chlorophyll *a*, chlorophyll *c*_2_, pheophytin *a*, β-carotene, diadinoxanthin, diatoxanthin and peridinin) and de-epoxidation state and (iv) oxidative stress biomarkers (e.g., DNA damage, lipid peroxidation, and catalase and superoxide dismutase activities).

## 2. Materials and Methods

### 2.1. Species Collection

The colony of scleractinian stony coral *Acropora* spp. (CITES 588369/02) was supplied and transported by commercial supplier TMC Iberia (Lisbon, Portugal) to Laboratório Maritimo da Guia facilities (Cascais, Portugal). The coral colony was fragmented into 29 pseudo-replicate fragments (average height 3.53 ± 0.99 cm) attached to clay tiles and (randomly) placed in an individual aquarium tank (4.2 L).

### 2.2. Acclimation Procedure and Hypoxia Exposure

After the acclimation time (7 days) under normoxic conditions (*n* = 29; O_2_ = 6.73 mg/L; T = 25.7 °C), the corals were exposed to two different experimental oxygen level treatments: (1) saturated O_2_ concentrations (control, *n* = 15; O_2_ = 6.73 mg/L, T = 25.7 °C) or (2) nocturnal hypoxia conditions (12 h) below the corals physiological threshold (hypoxia, *n* = 14; O_2_ = 1.75 mg/L, T = 25.7 °C) with daily normoxic conditions (*n* = 29; O_2_= 6.73 mg/L; T = 25.7 °C), that remained for 8 consecutive days of the experimental period.

Each coral fragment was placed in a separate aquarium tank and kept in a semi-open aquatic system. Natural seawater, pumped from the sea, was filtered (0.35 μm; Harmsco, Riviera Beach, FL, USA) and UV light sterilized (Vecton 150, TMC Iberia, Lisbon, Portugal) and was renewed daily (100–300 L). Aquarium tanks containing corals (control) were supplied by pumping (2200 w pump; TMC Iberia, Lisbon, Portugal) the seawater from the water outflow tank. However, seawater supplying the corals in the hypoxic treatment was pumped (3000 w pump; TMC Iberia, Lisbon, Portugal) from the water outflow tank to the cylindrical column and further to the designated tanks containing the corals. The cylindrical column tanks were injected with nitrogen gas with wooden air diffusers to remove oxygen synchronized with the night cycle (t = 12 h) and regulated by a solenoid valve connected to a timer. To maintain the O_2_ levels below 2 mg/L during the exposure, the injection of the nitrogen flow was manually adjusted and regulated with a pneumatic flow control valve (diameter 8 mm). The flow in all the individual tanks had been adjusted to 0.2 L/min and remained constant within all the experimental tanks. To ensure the stability of the water temperature, the experimental tanks containing the corals were placed in the water bath and the water temperature was controlled and kept steady by digital heaters (200 w; TMC Iberia, Lisbon, Portugal) and chillers (Frimar C250; Fernando Ribeiro, Lisbon, Portugal) recirculating water in the water outflow tank. To maintain the stability of water properties, a protein skimmer (V2skim Pro 450; TMC Iberia, Lisbon, Portugal) was installed in the water outflow tank with bioballs (matured with nitrifying bacteria) for biological quality (Ourico^®^; Fernando Ribeiro, Lisbon, Portugal). Throughout the experiment, oxygen concentrations had been constantly measured (in intervals of 1 s) with fibre-optic oxygen meter Firesting O_2_ (Pyro Science GmbH, Aachen, Germany) connected to the portable computer where the data had been recorded.

Light periodicity was 12 h night and 12 h day cycles at quantum irradiance of 300 μmol/ms (Aquaray lights; TMC Iberia, Lisbon, Portugal). Water physical and chemical parameters were monitored daily. Nitrates, nitrites, ammonium, phosphates, potassium, calcium and magnesium were measured daily with colourimetric tests (TMC Iberia, Lisbon, Portugal). The pH was measured using a manual measuring device (VWR pHenomenal^®^ pH 1100 H connected to an epoxy electrode (pH electrode DJ 113); VWR, Radnor, PA, USA), salinity with a refractometer (V2; TMC Iberia, Lisbon,, Portugal), carbonate chemistry (e.g., total alkalinity) was measured daily by spectrophotometry using the absorbance at 595 nm (Asys UVM 340 microplate reader, Biochrom Ltd., Cambridge, UK). To measure total alkalinity, formic acid and dye (bromophenol-blue) were added to the seawater to engage neutralization adapted from Sarazin et al. [48]. Temperature and oxygen were also monitored daily with a manual multi-parameter measuring device (WTW Multi 3510 IDS equipped with optical IDS dissolved oxygen sensors FDO^®^925; Weilheim, Germany). The daily measurements of seawater parameters can be found in Table S1.

### 2.3. Chlorophyll a Pulse Amplitude Modulated Fluorometry

On daily basis, Pulse Amplitude Modulated (PAM) chlorophyll fluorescence measurements were performed using PSI FluorPen MP 100-A (Photon System Instruments, Drásov, Czech Republic) [49,50,51] after dark-adapting the coral fragments for 15 min. A minimum of nine pseudo-replicates from each treatment was monitored for high data reliability due to replication effort [52,53]. The photochemical process was evaluated by the polyphasic rise in fluorescence (JIP-test) transient using OJIP protocol of the FluorPen and several photochemical parameters were extracted (Table 1). The JIP-test was performed daily in the afternoon (at 5:00 p.m.), and it comprises four phases. Level O represents all the open reaction centres at the onset of illumination with no reduction of Q_A_ (fluorescence intensity during 10 ms). The rise of transient from O to J indicates the net photochemical reduction of Q_A_ (the stable primary electron acceptor of PS II) to Q_A_^−^ (lasts for 2 ms). The phase from J to I is due to all reduced states of closed RCs such as Q_A_^−^ Q_B_^−^, Q_A_ Q_B_^2−^ and Q_A_^−^ Q_B_H_2_ (duration of 2–30 ms). The level P (300 ms) coincides with the maximum concentration of Q_A_−Q_B_ with a plastoquinol pool maximally reduced. Phase P also reflects a balance between the light incident at the PS II side and the rate of utilization of the chemical (potential) energy and the rate of heat dissipation [54]. JIP-test and LC1 test were performed using PSI FluorPen AP 150 (Photon System Instruments, Drásov, Czech Republic) on the last experimental day. Kautsky curves were obtained as results from JIP-test and rapid light curves (RLCs) from the LC1 test. The electron transport rate (ETR) at each light level and derived parameters were calculated with an absorptivity factor of 1 [52].

### 2.4. Coral Fragment Preparation and Homogenization Procedure

After the experimental period, each coral was fragmented using cutters (TMC Iberia, Lisbon, Portugal) and stored at −80 °C. Coral fragments were mixed in 1:5 (*w*/*v*) with K_2_HPO_4_/KH_2_PO buffer (pH 7.4) containing 1 mM DTT (dithiothreitol), 0.1 mM PMSF (phenylmethylsulfonyl fluoride), 1 mM EDTA (ethylenediaminetetraacetic acid) and 100 mM KCl (potassium chloride). To achieve tissue and cell disruption, sonification (Nanografi Nano Technology, City, Germany) was performed, and the homogenates were centrifuged at 1000× *g* for 10 s to ensure the complete separation of tissue from the skeleton. The resulting supernatant aliquots were used for oxidative stress and enzymatic antioxidant activity assays that were evaluated using a microplate reader (TECAN, Männedorf, Switzerland), and the reading was performed in triplicate.

### 2.5. Pigment Composition

Pigments capturing light are chlorophyll *a*, chlorophyll *c*_2_ and peridinin with pheophytin a and photoprotection pigments such as β-carotene, diadinoxanthin and diatoxanthin are distinctive for photosynthetic organisms [55]. Coral fragments for pigment analysis were extracted using 100% ice-cold acetone and subjected to an ultra-sound bath for 3 s to disrupt the symbiont pigment bearing cells. Extraction occurred for 24 h in the dark at −20 °C. After the extraction period, the resultant mix was centrifuged for 4000× *g* for 15 min at 4 °C, and the supernatant was used for spectral analysis in a dual-beam spectrophotometer. Absorbance was recorded against an acetone blank between 350 and 750 nm, at 0.5 nm steps and resultant data were analyzed using Gauss Peak Spectra fitting library, using SigmaPlot Software Kupper [56]. De-epoxidation state (*DES*) was used as an index to troubleshoot the accessory pigments in the xanthophyll cycle and was calculated as:

$$DES=\frac{Diatoxanthin}{Diatoxanthin+Diadinoxanthin}$$

### 2.6. Oxidative Stress and Antioxidant Enzyme Assay

DNA damage was assessed using DNA alkaline precipitation adapted from Olive [57] by monitoring DNA concentration in the remaining supernatant. Samples were mixed with 2% SDS consisting of 50 mM NaOH, 100 mM Tris base, 100 mM EDTA and 105 mL ultra-pure water. In addition, 0.12 M KCl was added to the mixture and incubated at 60 °C for 10 min and cooled in an ice bath for 15 min. Samples were centrifuged at 8000× *g* for 5 min at 4 °C. The supernatant was removed and 150 μL of DNA concentration was mixed with fluorescent Hoechst dye (150 μL, 1 μg mL^−1^ in 0.1 M K-phosphate buffer, pH 7.4), loaded into a microtiter plate (XPTO, non-binding), and fluorescence was measured at 360/460 nm of excitation and emission wavelength for 1 min using a Sinergy HT Microplate Reader (BioTek Instruments, Winooski, VT, USA). DNA damage (DNAd) was denoted as μg DNA per mg of total protein.

Lipid peroxidation (LPO) was evaluated according to Ohkawa et al. [58] as a measure of oxidative stress, whereby membrane lipid polyunsaturated fatty acid peroxides and thiobarbituric acid reactive substances (TBARS) degradation products react with 2-thiobarbituric acid (TBA). Samples (150 μL) were mixed with TCA 12%, TBA 0.73% Tris-HCl (pH 7.4), TBA 0.73% and 0.1 mM EDTA (150 μL). The samples were incubated at 97 °C for 60 min. Subsequently, they were cooled for 30 min and centrifuged at 13,400× *g* for 3 min at 25 °C. The sample was loaded into the 96-well microtiter plate and the concentration of TBARS was read at 535 nm (ε = 1.56 × 10^5^ M^−1^ cm^−1^) using the absorbance microplate reader. LPO was determined as nmol of TBARS produced per mg of total protein.

Total protein content followed the assay from Bradford [59], in which bovine serum albumin was used as a standard. Samples (4 μL sample tissue and 36 μL ultra-pure water) were added with 250 μL of Sigma Bradford solution into 96-well microtiter plates, and the absorbance was read at 595 nm with an absorbance microplate reader after 15 min of dark incubation at 25 °C. The calibration standard curve was obtained using dilution solution (0–10 μL standard protein solution bovine serum albumin (BSA) standards and 0–10 μL ultra-pure water) and Bradford solution (250 μL).

Catalase (CAT), an enzyme which protects the cell from ROS damage, was measured according to Aebi [60]. Hydrogen peroxide (150 μL 30 M H_2_O_2_ in 130μL 50 mM KH_2_PO_4_ buffer, pH 7) was added to each sample (20 μL) into the 96-well microtiter plate. The consumption of H_2_O_2_ and decrease in absorbance at 240 nm (ε = −39.4 mM^−1^ cm^−1^) was measured using the absorbance microplate reader. The CAT activity was compared with calibration and is denoted as μmol min^−1^ mg^−1^ of total protein.

Superoxide dismutase (SOD), another enzyme which protects the cell from ROS damage, was assayed following McCord and Fridovich [61] and modified according to Lima et al. [62] for 96-well microplate reading. SOD activity was determined using sample (10 μL), reaction mix (140 μL, 50 mM phosphate buffer (pH 7.8), 0.1 mM EDTA, 1.5 mM hypoxanthine, 0.15 mM cytochrome c), 30 mU ml^−1^ xanthine oxidase (60 μL) and the absorbance measured at 550 nm in a microplate reader. The reaction occurs when the xanthine oxidase/hypoxanthine system reduces the cytochrome c, indicating the inhibition of cytochrome c reduction by 50% by one unit of SOD enzyme. SOD activity was denoted as U mg^−1^ of total protein content.

### 2.7. Statistical Analyses

We used generalized linear models (GLM) with Gaussian or gamma family to determine the probability distribution of differences between the two treatment groups adopted from Zuur et al. [63]. Gamma family GLMs were used for rapid light curves variables (RLC) and Kautsky cuves (only M_0_, N, P_G_). Obtained values from each analysis have been analysed as response variables, and the treatment groups were used as predictor variables. The assumptions of the models (e.g., independence, normality and homogeneity of variance) were graphically verified [63]. Significance of the GLM was assumed when the probability was lower than 0.05 and was confirmed using Type-II test sum of squares using *Anova* function from the *car* package [64] in statistical software RStudio ver. 4.0.3 [65]. The results from the GLM statistical analyses for each variable are shown in Table 2.

## 3. Results

### 3.1. Photobiological Response

After the experimental period, no significant changes were observed in the maximum ETR (ETR_max)_, light saturation (E_K_), photosynthetic (α) and respiratory (β) efficiency derived from rapid light curves (Table 2 and Table S2) in corals exposed to hypoxia.

**Figure 1:**
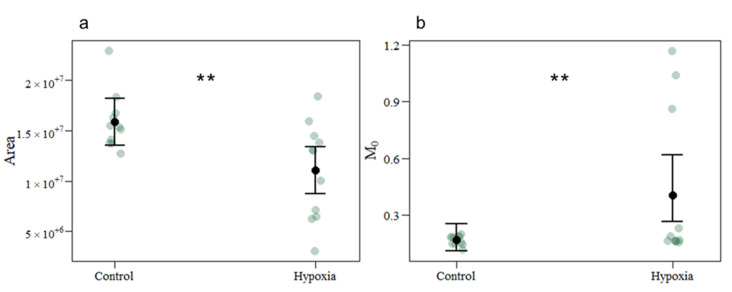
Extracted fluorescence parameters in coral fragments exposed to control and hypoxic conditions. (**a**) Oxidized quinone pool size (Area) and (**b**) net rate of PS II reaction centres closure (M_0_). The values represent mean and standard deviation, asterisks indicate significant differences between the treatment groups at: *p* < 0.01 (**) (control *n* = 11; hypoxia *n* = 11). The *n* denotes the number of coral samples assessed in the analysis. CI from the model and each measurement point are represented.

Observed fluorescence parameters (Figure 1) resulted in a significant reduction in oxidized pool size (Area) under hypoxic conditions. However, the reaction centre turnover rate (N) and the energy needed to close all reaction centres remained stable (S_M_). In contrast, the net rate of PS II reaction centres closure (M_0_) showed a significant increase under hypoxia.

**Figure 2:**
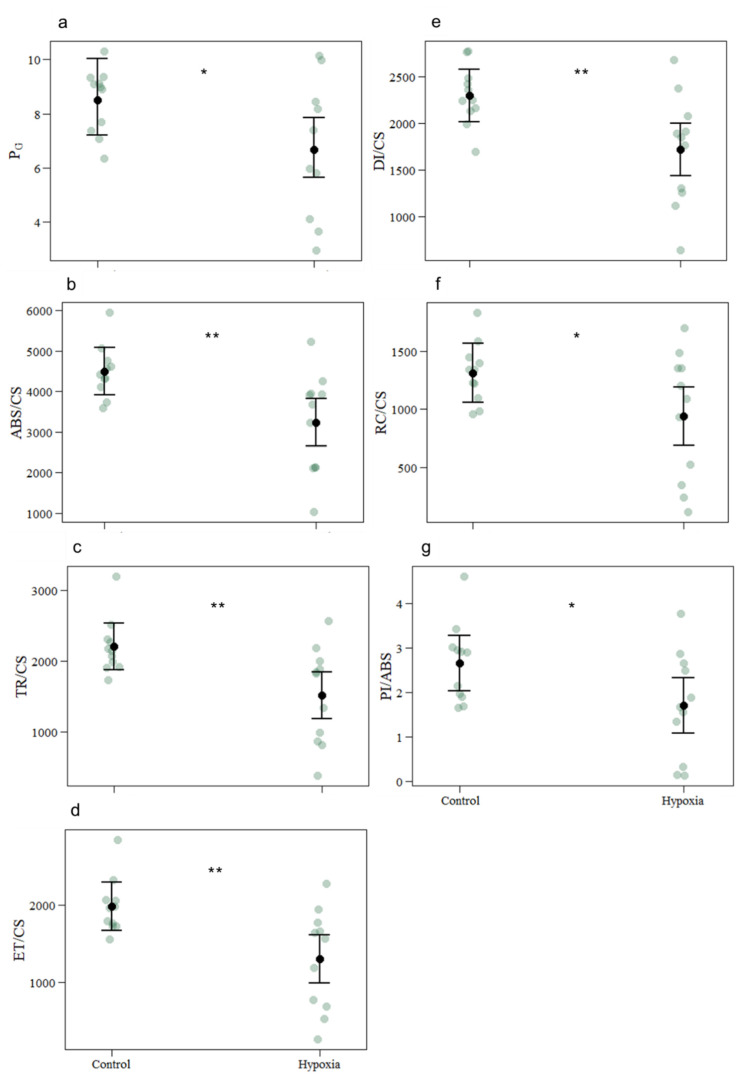
Photobiology-related phenomenological energetic parameters in coral fragments exposed to control and hypoxic conditions. (**a**) grouping probability between the two PS II (P_G_); (**b**) absorbed energy flux per cross-section (ABS/CS); (**c**) trapped energy flux per cross-section (TR/CS); (**d**) electron transport energy flux per cross-section (ET/CS); (**e**) dissipated energy flux per cross-section (DI/CS); (**f**) number of oxidized PS II reaction centres (RC/CS); (**g**) performance index (PI/ABS). The values represent mean and standard deviation, asterisks indicate significant differences between the treatment groups at: *p* < 0.05 (*); *p* < 0.01 (**) (control *n* = 11; hypoxia *n* = 11). The *n* denotes the number of coral samples assessed in the analysis. CI from the model and each measurement point are represented.

Contrarily to the values obtained from RLCs, several significant changes from JIP-test results (Figure 2) were detected (per cross-section). These values represent main phenomenological energetic fluxes that involve photochemical processes, from light-harvesting to its dissipation. There was a significant reduction in connectivity between the two PS II units (PG) and energy absorption by the PS II antennae (ABS/CS). Consistently with the ABS/CS results, the hypoxia induced a significant decrease in the effectively trapped energy flux in the PS II (TR/CS) and the energy flux transport in the electronic transport chain (ETC) (ET/CS). Concomitantly, a significant reduction was observed in energy dissipation flux (DI/CS) and the number of oxidized PS II reaction centres (RC/CS). The reaction centre density within the PS II antenna chlorophyll bed (RC/ABS) demonstrated no significant differences. Finally, the performance index (PI/ABS) exhibited a significant reduction under hypoxia.

### 3.2. Pigment Content

**Figure 3:**
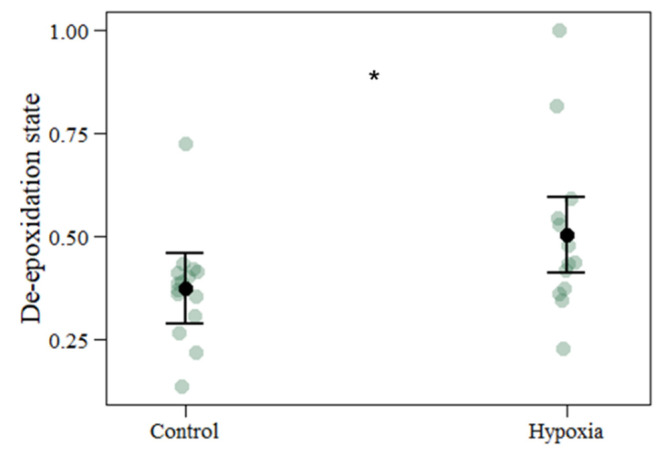
De-epoxidation state of coral fragments exposed to control and hypoxic conditions. The values represent mean and standard deviation, asterisks indicate significant differences between the treatment groups at: *p* < 0.05 (*) (control *n* = 15; hypoxia *n* = 13). The *n* denotes number of coral samples assessed in the analysis. CI from the model and each measurement point are represented.

Pigment analyses (Table 2 and Table S3), performed as an oxidative stress biomarker, revealed no significant differences between the hypoxic and control treatment group. The pigment assay involved the quantification of chlorophyll *a*, chlorophyll *c*_2_, pheophytin *a*, β-carotene, diadinoxanthin, diatoxanthin and peridinin. However, the de-epoxidation state (Figure 3), entailing conversion from diadinoxanthin to diatoxanthin, was significantly increased under the overnight hypoxia treatment.

### 3.3. Oxidative Stress Biomarkers

**Figure 4:**
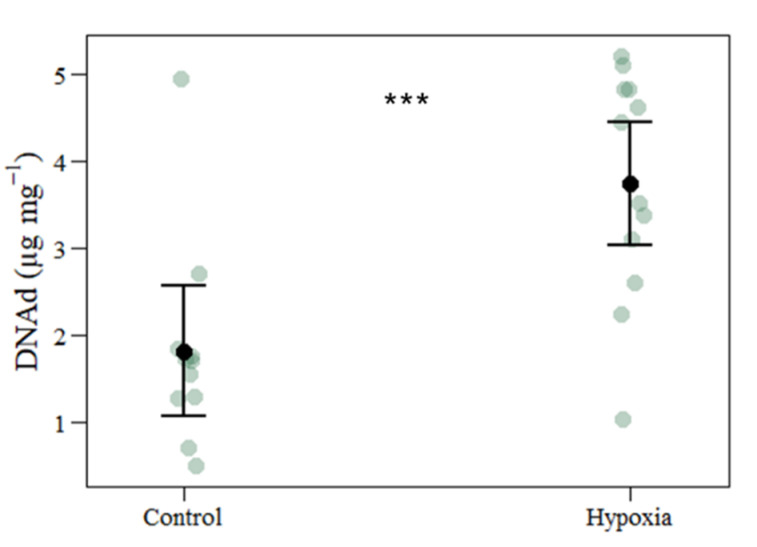
Oxidative DNA damage caused by ROS of coral fragments exposed to control and hypoxic treatments. The values represent mean and standard deviation, asterisks indicate significant differences between the treatment groups at: *p* < 0.001 (***) (control *n* = 12; hypoxia *n* = 12). The *n* denotes the number of coral samples assessed in the analysis. Abbreviations: µg: microgram; mg: milligram. CI from the model and each measurement point are represented.

Regarding cellular damage (Table 2), there was a significant increase in DNA damage (Figure 4) under hypoxia, while the LPO demonstrated no changes among the treatment groups. Protein content was assessed as enzymatic antioxidant response (Table 2) to disclose the potential defence mechanisms against abiotic stress; however, no differences were observed between the two treatment groups. Considering SOD and CAT activities (Table 2 and Table S4), there were no changes between the control and hypoxia treatments.

## 4. Discussion

Severe decreases in nocturnal DO concentrations have been documented in Australia due to warming, which decreases oxygen solubility and increases the rate of biological oxygen consumption. In certain regions of the Caribbean, it is also known that oxygen levels may go below 2 mg/L as a result of anthropogenic eutrophication and nutrient input, and these hypoxic episodes usually last several days [10,15,17,36,43,66]. *Acropora* coral species are known to exhibit high susceptibility to climate change, including ocean warming and deoxygenation [10,15,27,34,67,68]. Symbiodiniaceae produce oxygen that corals utilize to maintain sufficient oxygen concentrations in their tissue and surrounding of the coral. The coral-algal interaction enables corals to cope with decreased oxygen concentrations to a certain extent by the nutrient and metabolite exchange [10,26].

Symbiodiniaceae provide corals with most of the required energy by producing ATP from NADPH at the light-harvesting complexes through electron transport chain (ETC) between photosystem II (PS II) and photosystem I located in the chloroplasts thylakoid membrane [69]. Previous studies found that the oxidative stress that emerges from hypoxia induces a decrease in photosynthetic electron transport and PS II reaction centres’ efficiency. Hence, photosynthetic defence mechanisms are responsible for preventing excess excitation energy accumulation, which is the source of high ROS production that damages lipids, proteins and pigments [70,71].

The present study assessed the effects of nocturnal hypoxia in photochemical energy fluxes at the oxygen-evolving complexes. While there were no changes in photosynthetic (α) or respiratory (β) efficiency, the photochemical activity reduction was caused by the impairment of the electron transport and thus a decrease in the electron transport energy flux, as shown by the significant change in connectivity between the PS II antennae (P_G_) in samples exposed to nocturnal hypoxia [52,72]. Decreased connectivity has cascading effects on subsequent electron transport due to reduced PS II antennae energy transduction. This was evident in the phenomenological energy fluxes measured per excited cross-section (CS) of the photosynthetic symbionts [53]. The reduction in the pigment antenna absorption of the photon flux (ABS/CS) and the decrease in the energy dissipation (DI/CS) led to an insufficient amount of transported energy to the reaction centres (ET/CS). Despite that, the number of transferred electrons in the ETC (N) was unchanged. Nonetheless, there was an increase in the demand for light-harvesting to tackle the remaining energy depletion [73]. Following the diminished absorption energy flux, the reduced reaction centres (RC/CS) density explains the lowered amounts of energy absorbed and trapped by the PS II [54,74]. An increase in the net rate of closure of all PS II RC (M_0_) could account for light absorption energy flux preservation. The efficiency of the harvested energy could be explained by the decrease in reaction centre density and trapped electrons at PS II (TR/CS) [52]. Regardless, the change in energy required to close all the RCs (S_M_) was unaltered. However, the reduction in oxidized quinone pool size (Area) supports the decrease in the electron transport energy flux, which involves electron transport from plastoquinol to end receptors of PS I [54]. Due to the reduced transport of excessive energy with limited oxidized quinone pool size, this could have altered PS II efficiency [73]. Furthermore, the reduced need for redox potential dissipation produced during light-harvesting is due to the lowered energy fluxes [52]. According to Ulstrup et al. [75], symbiotic malfunction of the photosynthetic apparatus of *Pocillopora damicornis*, when subjected to 0% air-saturation, demonstrated similar photochemical responses to those exhibited from the decline and changes in the JIP-test values. This could be a result of a reduction in electron receptors of PS II, the cycle of the electron transport encompassing the PS I, or photorespiration.

Hypoxic conditions may affect the behaviour and function of PS II on account of the decreased trapped energy amounts and lower energy flux to the ETC, followed by less excitation energy dissipation and lower performance index (PI/ABS) [76]. As suggested by Finelli et al. [77], depleted oxygen concentrations (0.3% air-saturation) in an environment without flow lead to a decrease in photosynthetic activity (caused by the excess photo dissipation from photorespiration) in the coral *Agaricia agaricites*. Mass et al. [78] also support this by showing that photorespiration impeded photosynthesis in the coral *Favia veronica*, the red alga *Gracilaria cornea* and the seagrass *Halophila stipulacea* under lowered oxygen conditions.

In the present study, pigment composition assays did not exhibit any differences in the contents of chlorophyll *a*, chlorophyll *c*_2_, peridinin that are typical for Symbiodiniaceae [42], as well as for β-carotene, diadinoxanthin, diatoxanthin and pheophytin *a* under both treatments. Among these pigments, carotenoids are known to act as ROS quenchers in regard to their photoprotective functions [40,79]. Their involvement in the antioxidant activity, xanthophyll cycle and quenching of ^1^O_2_ constitute the protection against oxidative stress, with β-carotene having the highest relevance in ROS scavenging [69,80,81]. Carotenoid pool de-epoxidation state as a photoprotection mechanism against photo-oxidative damage defends the thylakoid membrane in the chloroplasts when the excitation energy at PS II exceeds the ETC capabilities and increases the non-photochemical quenching [60,72]. This process involves the conversion of diadinoxanthin to diatoxanthin in order to dissipate underutilized energy and release its excess as heat by xanthophyll pigments during the de-epoxidation reaction [73,81,82]. Here, we showed an observable increase in carotenoid content in the hypoxic treatment group.

The production of ROS is capable of inducing cellular damage, should the coral defence system fail to suppress their production and quench the accumulation [37,83,84]. Hydrogen peroxide (H_2_O_2_) is an oxygen reactive intermediate that can harm nuclear and plastidial DNA. Breakdown of DNA results in mutations, deletions and other genetic complications due to Fenton reactions that produce hydroxyl radical as opposed to H_2_O_2_ and O_2_^−^ [84,85]. In the present study, DNA damage was notably higher under hypoxia, in opposition to the unaltered results obtained from the antioxidant enzyme activity. This may be a consequence of the ROS build-up in the intracellular space that triggers DNA degradation.

Another threat to cellular function is oxidative damage, such as lipid peroxidation (LPO), which deteriorate to aldehydes and hydrocarbons when the PS II is over-excited and excess energy accumulates, prompting the production of ROS, namely superoxide radicals [69,84,86]. However, the LPO assay did not reveal any differences between treatments, which is in accordance with the antioxidant defence responses. Moreover, oxidative stress is known to inflict the destruction of proteins, leading to deformation of amino acids, peptide chain fragmentation, electrical charge alteration and an increase in the predisposition to dispose and degrade amino acids [84,85]. Exposure to nocturnal hypoxia can increase the activity of the defence system of enzymatic antioxidants as an effective strategy to overcome increased ROS production. Superoxide dismutase (SOD) is linked to the antioxidant defence mechanisms, which is responsible for the catalysis of superoxide radicals. The latter promote the free radical oxidation chain that eventually leads to the inactivation of enzymes and the formation of other ROS. The main reason for increased ROS production is accelerated mitochondrial respiration rate since the hypoxia limits the oxygen supply [87]. However, another source of superoxide radical lies in the electron acceptor of PS I [69]. Such an increasing trend in SOD activity was not detected in this study, which is consistent with Teixeira et al. [88], suggesting that mitochondrial damage was absent. In contrast, the mitochondrial damage would cause decreased respiration and superoxide radical production during the oxidative phosphorylation process located in the cytochrome pathway of the mitochondrial ETC [69]. There is evidence of higher cellular reoxygenation injury in *Acropora nobilis* and *Alveopora verrilliana*, while the SOD activity was lower when exposed to hypoxia. In addition, seemingly the SOD activity is positively associated with chlorophyll content in symbiotic anemones *Anthopleura elengantissima*, because photosynthetic production of O_2_ and O^−^_2_ in zooxanthellae regulates SOD activity [89]. These prior studies support the findings of this study, where SOD and chlorophyll content did not differ under oxidative stress.

To preserve symbiosis, it is necessary for catalase (CAT) activity to have a high sensitivity to both oxidative and temperature stress [84]. CAT activity showed no variation between treatments, which is consistent with the LPO levels, chlorophyll concentrations and SOD activity. While there is a strong correlation of antioxidant enzyme responses and chlorophyll content, other enzymes (i.e., peroxidases), antioxidants (i.e., ascorbic acid, glutathione, tocopherol and carotenoids) and fluorescent proteins could prevent the deleterious effects of ROS as antioxidant defence mechanisms [84,86].

## 5. Conclusions

The findings of this study suggest that the coral *Acropora* spp. exhibited resilience to nocturnal low oxygen conditions. Indeed, corals showed an increase in oxidative stress with observable impacts on the photosynthetic apparatus functioning and DNA. Different metabolic and physiological processes are executed by cells of the host and symbionts to limit stress and prevent death due to ROS production and accumulation. However, the antioxidant defence system was not stimulated under hypoxic stress conditions in this study. However, it is important to note that corals could have exceed the threshold of physiological performance under more prolonged hypoxic conditions. Climate change can severely affect coral reefs, and, with decreasing oxygen concentration trends and more frequent hypoxic events, there is a high probability of losing a massive part of coral reefs in the Anthropocene [25,32]. Coral bleaching is a biological indicator of the extreme environmental perturbation effects related to climate change driven oxygen oscillations that force many marine species towards their survival threshold and hence reduce their ability to develop the crucial tolerance [23,90].

## Figures and Tables

**Table 1:** Summary of the extracted fluorometric parameters in the analysis.

Variable	Description
**Rapid light curves**	
ETR_max_	Maximum ETR obtained from the RLC
E_K_	The onset of light saturation
α	Photosynthetic efficiency extracted from the RLC initial slope
β	Respiratory efficiency
**Kautsky curves**	
Area	Oxidized quinone pool size available for reduction and is a function of the area above the Kautsky plot
S_M_	The energy needed to close all reaction centres
M_0_	The net rate of PS II reaction centres closure.
N	Reaction centre turnover rate
P_G_	Grouping probability between the two PS II units
ABS/CS	Absorbed energy flux per cross-section.
TR/CS	Trapped energy flux per cross-section.
ET/CS	Electron transport energy flux per cross-section.
DI/CS	Dissipated energy flux per cross-section
RC/CS	The density of available reaction centres (Q_A_-reducing PS II reaction centres) per cross-section
RC/ABS	The density of the reaction centres II within the PS II antenna chlorophyll bed
PI/ABS	Performance index on equal absorption basis

**Table 2:** GLM outcomes for all the analysed variable responses of corals exposed to control and hypoxic treatment. Statistical significance at *p*-value < 0.05 in bold. Abbreviations: SE: standard error.

Variable	Mean (Control)	Mean (Hypoxia)	SE	Estimate	t-Value	*p*
**Rapid light curves**						
ETR_max_	15.3333	18.6364	0.3804	0.1951	0.513	0.608
E_K_	475.8107	313.3652	0.3994	−0.4177	−1.046	0.299
α	0.0422	0.0432	0.1079	0.0223	0.206	0.837
β	0.1885	0.1811	0.0625	−0.0403	−0.644	0.520
**Kautsky curves**						
Area	15,891,063.4545	11,075,497.2727	167,746	−4,815,566	−2.871	**0.004**
S_M_	3696.1655	4012.1709	181.9	316	1.738	0.082
M_0_	0.1676	0.4065	0.3021	0.8859	2.932	**0.004**
N	6.2543	8.1671	0.1634	0.2668	1.633	0.103
P_G_	8.5090	6.6717	0.1182	−0.2433	−2.058	**0.040**
ABS/CS	4498.2727	3237.8182	420.3	−1260.5	−2.999	**0.003**
TR/CS	2201.4657	1520.1228	236.4	−681.3	−2.882	**0.004**
ET/CS	1982.7497	1303.6097	223.8	−679.1	−3.035	**0.002**
DI/CS	2296.8070	1717.6954	201.3	−579.1	−2.876	**0.004**
RC/CS	1311.6541	940.8176	182.1	−370.8	−2.036	**0.042**
RC/ABS	0.3226	0.3094	0.0273	−0.0132	−0.482	0.630
PI/ABS	2.6528	1.7112	0.4471	−0.9416	−2.106	**0.035**
**Pigment composition**						
Chlorophyll *a*	0.0041	0.0035	0.0004	−0.0007	−1.735	0.083
Chlorophyll *c*_2_	3.85 × 10^−5^	3.2 × 10^−5^	1.02 × 10^−5^	−6.54 × 10^−6^	−0.640	0.522
Pheophytin *a*	0.0040	0.0043	0.0006	0.0003	0.459	0.647
β-carotene	0.0041	0.0037	0.0005	−0.0005	−1.010	0.313
Diadinoxanthin	1.61 × 10^−4^	1.29 × 10^−4^	3.07 × 10^−5^	−3.27 × 10^−5^	−1.065	0.287
Diatoxanthin	1.07 × 10^−4^	1.41 × 10^−4^	3.50 × 10^−5^	3.37 × 10^−5^	0.963	0.336
Peridinin	1.9686	2.3235	0.3001	0.3549	1.183	0.237
De-epoxidation state	0.3721	0.5037	0.0640	0.1316	2.057	**0.039**
**Oxidative stress**						
DNA damage	1.8168	3.7431	0.5264	1.9263	3.660	**0.001**
Lipid peroxidation	1.8796	2.0588	0.2884	0.1792	0.621	0.534
Total protein	2.5062	2.7733	0.2004	0.2672	1.333	0.183
Catalase	14.3298	18.2601	2.882	3.93	1.364	0.173
Superoxide dismutase	0.5275	0.4973	0.0466	−0.0302	−0.649	0.516

## Data Availability

The statistical analysis code script and dataset are available in the repository, 10.6084/m9.figshare.19077413.

## Supplementary Materials

The following are available online at https://www.mdpi.com/article/10.3390/biology11071068/s1, Table S1: Seawater physicochemical parameters in all experimental setups. Table S2: Analysis of deviance table (Type II tests) for the generalized mixed models for photobiological responses of corals exposed to control and hypoxic treatment. Table S3: Analysis of deviance table (Type II tests) for the generalized mixed models for pigment composition analysis of corals exposed to control and hypoxic treatment. Table S4: Analysis of deviance table (Type II tests) for the generalized mixed models for oxidative stress biomarker analysis of corals exposed to control and hypoxic treatment.
