# Supplementary material for: Effects of Hypoxia on Coral Photobiology and Oxidative Stress
Source: Biology (Basel). 2022 Jul 18;11(7):1068. doi: 10.3390/biology11071068 (PMC9312924; doi:10.3390/biology11071068)
Supplement: Supplementary file 1 [file biology-11-01068-s001.zip › biology-1621696-supplementary.pdf]

Supplemental materials

Effects of Hypoxia on Coral Photobiology and Oxidative Stress

Mark Deleja, José Ricardo Paula, Tiago Repolho, Marco Franzitta, Miguel Baptista, Vanessa Lopes, Silvia Simão, Vanessa F. Fonseca, Bernardo Duarte and Rui Rosa

Table S1. Seawater physicochemical parameters in all experimental setups.

	Control	Hypoxia
Temperature (ºC)	25.7 ± 0.3	25.7 ± 0.3
Salinity (ppt)	35	35
pH	8.07 ± 0.02	8.07 ± 00.2
TA	0.61 ± 0.03	0.61 ± 0.03
Oxygen day (mg O_2/l)	6.73 ± 0.12	1.75 ± 0.1
Oxygen night (mg O_2/l)	6.73 ± 0.12	6.73 ± 0.12
NO_2 (ppm)	0.05	0.05
NO_3 (ppm)	0	0
NH_{4+} (ppm)	0.05	0.05
PO_4 (ppm)	0.03	0.03
K^+ (ppm)	422.7917 ± 13.41	421.1786 ± 12.54
Ca (ppm)	447.9 ± 32.81	445.0714 ± 16.44
Mg (ppm)	1326 ± 26.64	1328.3 ± 25.3

Physiochemical parameters were measured daily in triplicates and averaged per individual and per treatment over the experimental period. The measurements are represented as a mean value with standard error (mean ± std. error).

Table S2. Analysis of deviance table (Type II tests) for the generalized mixed models for photobiological responses of corals exposed to control and hypoxic treatment. Statistical significance at p-value < 0.05 in bold.

	df	x^2	p
**Rapid light curves**			
ETR_{max}	1	0.263	0.608
E_K	1	1.078	0.299
α	1	0.043	0.837
β	1	0.415	0.520
**Kautsky curves**			
Area	1	8.241	**0.004**
N	1	2.658	0.103
S_M	1	3.019	0.082
M_0	1	8.330	**0.004**
P_G	1	4.226	**0.040**
ABS/CS	1	8.993	**0.003**
TR/CS	1	8.307	**0.004**
ET/CS	1	9.211	**0.002**
DI/CS	1	8.273	**0.004**
RC/CS	1	4.146	**0.042**
RC/ABS	1	0.232	0.630
PI/ABS	1	4.435	**0.035**

Table S3. Analysis of deviance table (Type II tests) for the generalized mixed models for pigment composition analysis of corals exposed to control and hypoxic treatment. Statistical significance at p-value < 0.05 in bold.

	**df**	**x^2**	**p**
Chlorophyll *a*	1	3.009	0.083
Chlorophyll c_2	1	0.409	0.522
Pheophytin *a*	1	0.210	0.647
β-carotene	1	1.020	0.313
Diadinoxanthin	1	1.133	0.287
Diatoxanthin	1	0.927	0.336
Peridinin	1	1.399	0.237
De-epoxidation state	1	4.231	**0.039**

Table S4. Analysis of deviance table (Type II tests) for the generalized mixed models for oxidative stress biomarker analysis of corals exposed to control and hypoxic treatment. Statistical significance at p-value < 0.05 in bold.

	**df**	**x^2**	**p**
DNA damage	1	13.393	**0.001**
Lipid peroxidation	1	0.386	0.534
Total protein	1	1.777	0.183
Catalase	1	1.860	0.173
Superoxide dismutase	1	0.421	0.516
